# Generation of a Movement Scheme for Positive Training

**DOI:** 10.3389/fnins.2017.00096

**Published:** 2017-03-01

**Authors:** Lin Liu, Le Xie, Yun-Yong Shi, Bing-Chen An

**Affiliations:** ^1^Institute of Forming Technology and Equipment, Shanghai Jiao Tong UniversityShanghai, China; ^2^School of Biomedical Engineering, Shanghai Jiao Tong UniversityShanghai, China; ^3^HuaDong Hospital Affiliated with Fudan UniversityShanghai, China

**Keywords:** rehabilitation, training, upper limb, robot, trajectory

## Abstract

Rehabilitation robots have been demonstrated to be an efficient tool in the field of rehabilitation training. Meanwhile, there are varieties of tasks designed for motion training. These tasks need to be transmitted to motion data for rehabilitation robots. In this paper, we designed a drinking task and captured the motion data as the ground truth, through sensors of an exoskeleton device named Neo-Arm. To verify the effectiveness of Neo-Arm, we used a Vicon system to capture the same motion task without Neo-Arm for comparison. Eight subjects participated in the experiment. The motion data of the drinking task, including the range of motion (ROM) and the velocity of each joint, are obtained. The result shows that the Neo-Arm can achieve the suitable precision and be fit for other kinds of upper limb motion tasks.

## Introduction

A stroke, also called a cerebral vascular accident (CVA), is one kind of acute disease in the blood vessels in the brain characterized by varying degrees of neurological deficit, which leads to series of sequelae and symptoms. Among all the symptoms, hemiplegia is the most common. However, it is quite difficult for hemiplegic patients to regain their motion function of the upper limb, which leads to a difficult life with a constant reliance on family assistance and medical care. Hence, a variety of rehabilitation robots have been developed to help hemiplegic patients receive better rehabilitation training.

Early studies on rehabilitation robots are mainly based on the design using end-effecter mechanisms. For example, Stanford university developed an upper limb rehabilitation robot called MIME (Lum et al., 2004, 2006), based on the PuMA550 robot. Meanwhile, MIT-MANUS (Krebs et al., 2004), GENTLE/S (Coote et al., 2008), and iPAM (Holt et al., 2007) were developed. Although, these robots can lead the arm to move in space with multiple degree of freedom (multi-DOF) movements, they are not capable of controlling each joint of the upper limb during each period of motion. To control the upper limb from each joint, a variety of rehabilitation robots were designed as exoskeleton mechanisms, such as T-WREX (Housman et al., 2007), Intelliarm (Park et al., 2008), MEDARM (Ball et al., 2007), RUPERT (He et al., 2005), Dampace (Stienen et al., 2009), SUEFUL-7 (Gopura et al., 2009), and Armin I, II, and III (Nef and Riener, 2005; Mihelj et al., 2007; Nef et al., 2009). Nevertheless, they are not suitable for capturing motion information from active movement. Because these robots are heavy for subjects, motors, and corresponding drive mechanisms add more weight to the robots. In this case, subjects have to use more strength and change how they move to offset the weight and the inertia of the mechanism.

To capture movement, optical measurement equipment has been widely used, which can record the trajectory of the marked point in the skin of the subject, such as Kinect (Chang et al., 2013; Uttarwar and Mishra, 2015) and Vicon (Chung et al., 2011; Henmi et al., 2014).

However, there is relative motion between the skin and inner bones during the upper limb movement, especially for internal/external rotation, which can lead to angular errors. Meanwhile, upper limb movement is complicated with multi-DOFs in 3-D space, which can lead to a blocking problem when optical equipment is used to capture movement trajectories.

The objective in this research is to capture healthy people's trajectory in a motion task using an upper limb exoskeleton device. The tasks was designed as a series of movements for drinking. Eight healthy subjects participated in the experiment. They were instructed to wear Neo-Arm to perform the drinking task and to perform the same task using the Vicon system for comparison. During the task, angular information was recorded by both Neo-Arm and the Vicon system. Finally, the 3D trajectory of the upper limb movement was obtained.

## Materials and methods

### Device description

#### Neo-Arm

**Figure 1:**
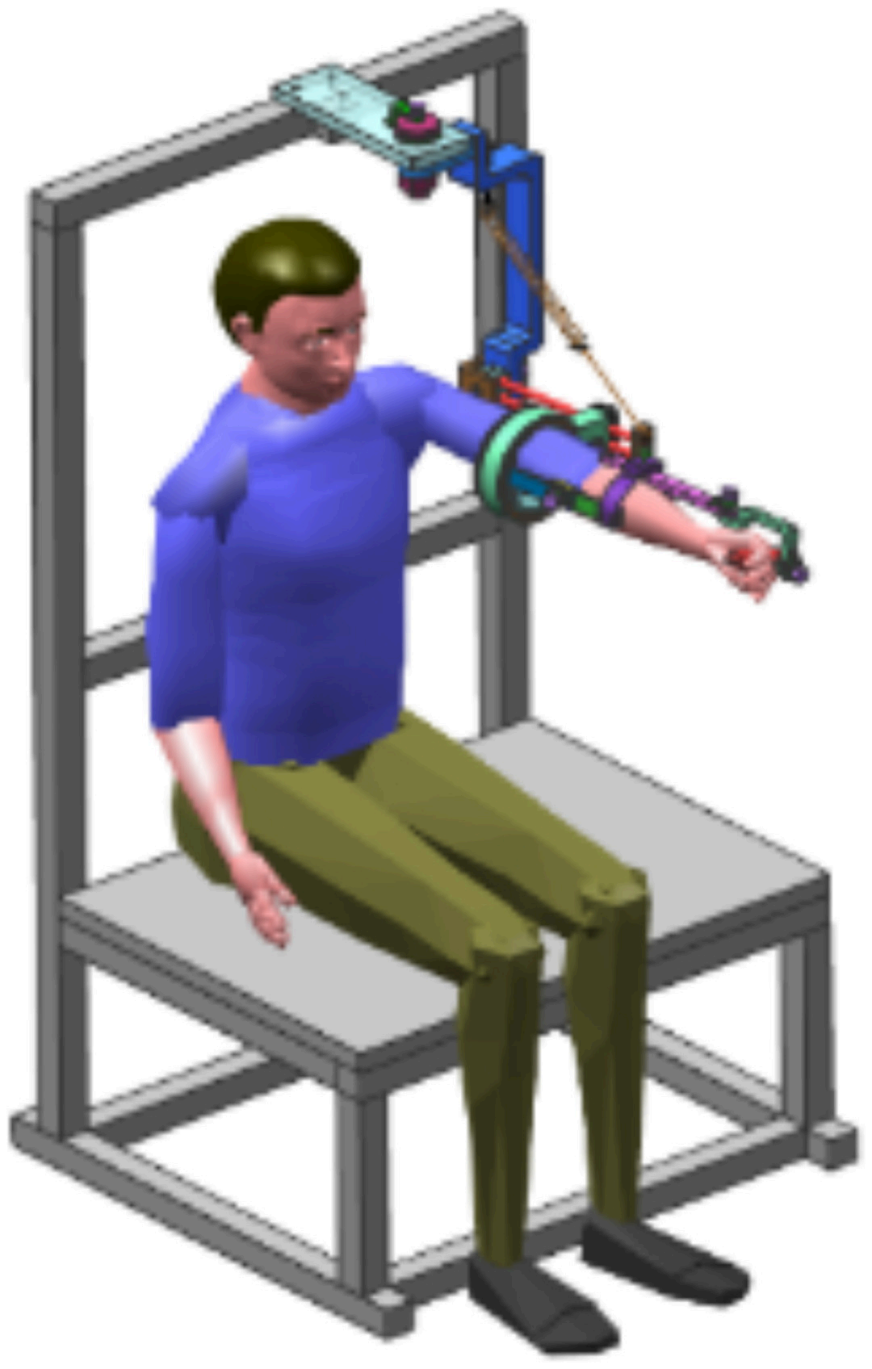
**Neo-Arm device**.

A 7-DOF rehabilitation device for active training was developed in our earlier research (Yu et al., 2015), called the Neo-Arm device, as shown in Figure 1. The device is equipped with an angular displacement transducer at each DOF, which can capture the position information of real-time movement. Neo-Arm can be used to capture the 3D trajectory of the rehabilitation movement in positive training.

**Figure 2:**
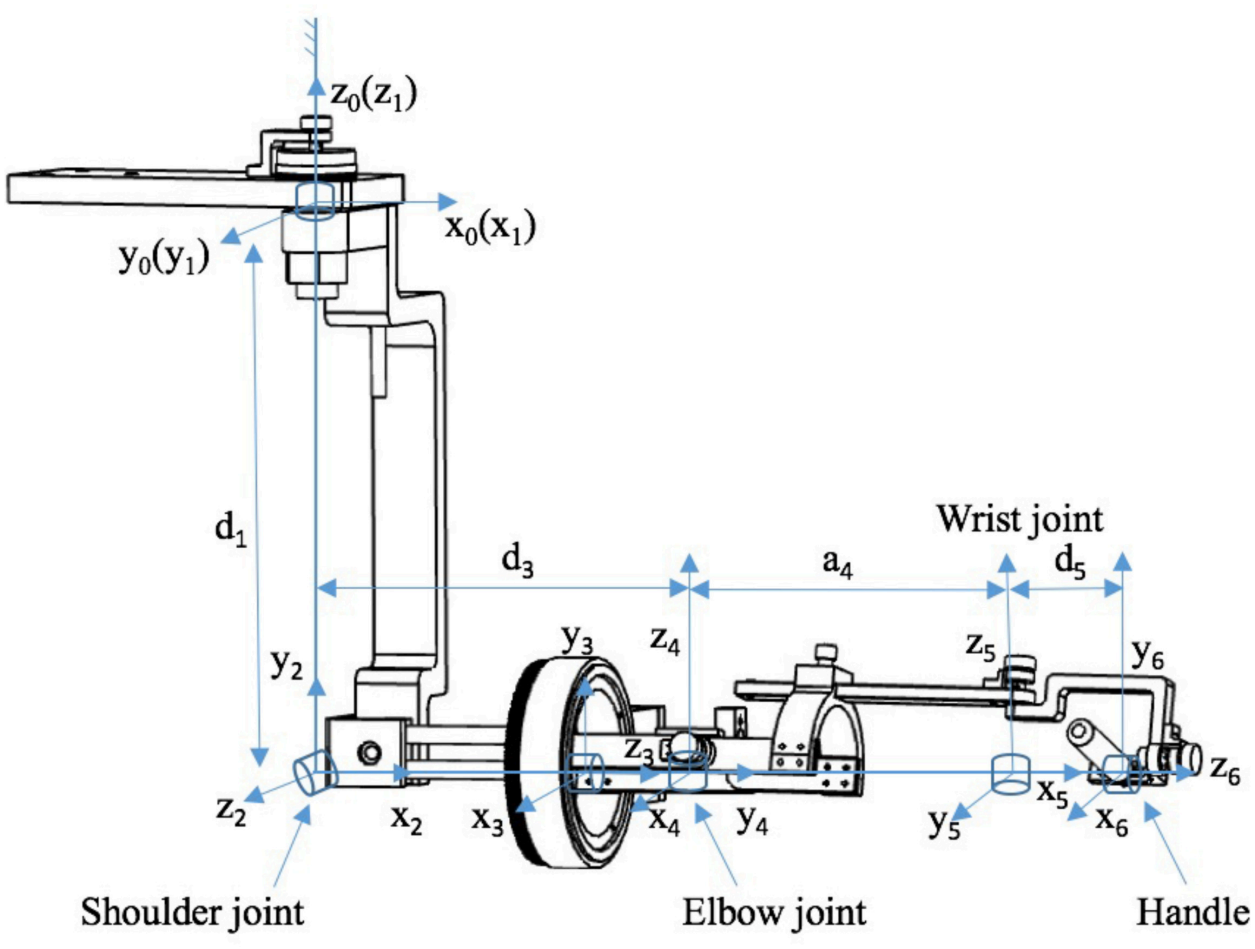
**Coordinate system of the Neo-Arm robot**.

At each joint of Neo-Arm, an angular displacement sensor is equipped through the axis of the rotation, by which the angular information can be captured and transformed from electrical signals to angular data. Through the training system, data are saved as a file for the subject. The coordinate system of Neo-Arm is shown in Figure 2. Through the coordinate system and the data captured by sensors, we can calculate the 3D trajectory of the endpoint of the upper limb.

The Denavit–Hartenberg (D–H) matrix is adopted as Equation (1):

(1) T60=10T21T32T43T54T65T=[nxoxnyoyaxpxaypynzoz00azpz01].

where, {*p*_*x*_ *p*_*y*_ *p*_*z*_}^*T*^ is the location of the endpoint of the robot.

Table 1 is also needed as the demand of D–H matrix to calculate the endpoint position of the robot in 3D space.

**Table 1:** **D-H table**.

***I***	**θ_*i*_**	**a_*i*−1_**	**d_*i*_**	**α_*i*−1_**
1	θ_1_	0	d_1_	0
2	θ_2_	0	0	π/2
3	θ_3_	0	d_3_	π/2
4	θ_4_	0	0	π/2
5	θ_5_	a_4_	0	π/2
6	θ_6_	0	d_5_	π/2

In Table 1, the values of *d*_*i*_ and *a*_*i*−1_ are the length parameters of Neo-Arm. The length parameters for upper limb and Neo-Arm are shown in Table 2.

**Table 2:** **Length parameters of the upper limb and Neo-Arm**.

**Definition**	**Upper limb**	**Neo-Arm**
Distance between the base point and shoulder joint	L1	d_1_
Length of the upper arm	L2	d_3_
Length of the forearm	L3	a_4_
Distance between the elbow joint and handle	L4	d_5_

In Table 1, the values of θ*i* are the angle parameters of the DOF of the human upper limb. The values of J_i_ can be captured by an angular displacement sensor. The angle parameters for the upper limb and Neo-Arm are shown in Table 3.

**Table 3:** **Angle parameters of the upper limb and Neo-Arm**.

**Joints of upper limb**	**DOF of upper limb**	**Angle of upper limb**	**Angle of Neo-Arm**
Shoulder	Flexion/extension	θ_1_	J1
	Abduction/adduction	θ_2_	J2
	Internal rotation/external rotation	θ_3_	J3
Elbow	Flexion/extension	θ_4_	J4
Forearm	Pronation/supination	θ_5_	J5
Wrist	Flexion/extension	θ_6_	J6
	Ulnar deviation/radial deviation	θ_6_	J6′

#### Vicon system

**Figure 3:**
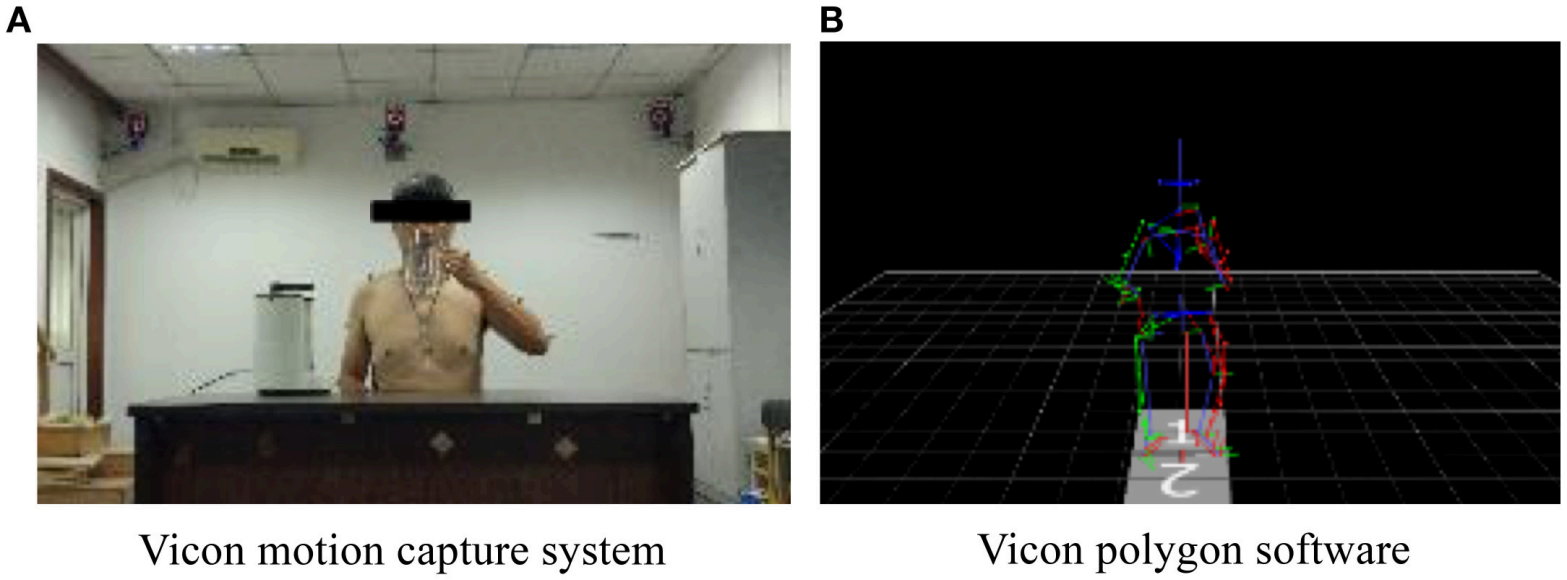
**The motion captured in the Vicon system. (A)** Vicon motion capture system. **(B)** Vicon polygon software.

The optional motion capture system used in the experiment is a Vicon system (Oxford Metrics Ltd., Oxford, UK), in Ruijin Hospital, Shanghai Institute of Orthopedics and Traumatology, Shanghai, China, see Figure 3A. It is composed of 10 cameras and an MX13 motion capture system. The body of the subject is marked with 39 markers (14 mm in diameter), which are used to record trajectories in a sampling frequency of 100 Hz. The motion data are processed in Vicon polygon software, see Figure 3B.

### Subjects

Eight male subjects were selected to participate in the experiment. This study was carried out in accordance with the recommendations of Ethics Committee at Shanghai Ninth People's Hospital affiliated to Shanghai Jiao Tong University School of Medicine (No. 2013011). All subjects provided written informed consent prior to participation. Subjects are similar in age and sitting height, as well as their upper limb dimensions, as described in Table 4.

**Table 4:** **Subjects**.

**Subjects**	**Sex**	**Age**	**Weight (kg)**	**Height (cm)**	**L2 (mm)**	**L3 (mm)**	**L4 (mm)**
1	M	32	75	175	358	272	73
2	M	27	68	173	359	270	72
3	M	25	71	171	357	268	72
4	M	25	70	173	356	268	75
5	M	30	80	175	359	271	72
6	M	26	79	182	355	279	71
7	F	26	71	172	356	276	70
8	F	28	66	170	355	272	72

Meanwhile, the dimensions of the Neo-Arm device can be adjusted to accommodate the distinctive dimensions of the upper limb for different subjects, by which means each joint of the whole mechanism can be correlated with the corresponding joint of the subject's limb joint. The dimension information can be saved as a record. The basic parameters of the device are shown in Table 5.

**Table 5:** **Neo-Arm device parameters**.

**L1**	**L2**	**L3**	**L4**
435 mm	360 mm	270 mm	70 mm

### Task design requirement

The activities of daily living (ADL) include various self-care activities people perform in everyday lives, such as bathing, feeding, drinking, and dressing. Since the assessment of ADL has played an important role in rehabilitation training, it is significant that in this research subjects should follow the mode of motion in ADL. Given that drinking is a basic movement performed repeatedly in everyday life, drinking was proposed and discussed as a task in this paper.

To simulate drinking in daily life, the task was designed based on three principles. First, each time subjects should perform a different movement. Second, the movement that subjects perform should be a motion combination including picking up a cup, fetching water, and drinking. Third, the ROM of the device should be within the ROM demanded in the task. Different movement trajectories combined with motion combinations can avoid the simple advance and return movement in rehabilitation training. In this way, subjects are led to finish the task by actively thinking.

### Equipment layout

**Figure 4:**
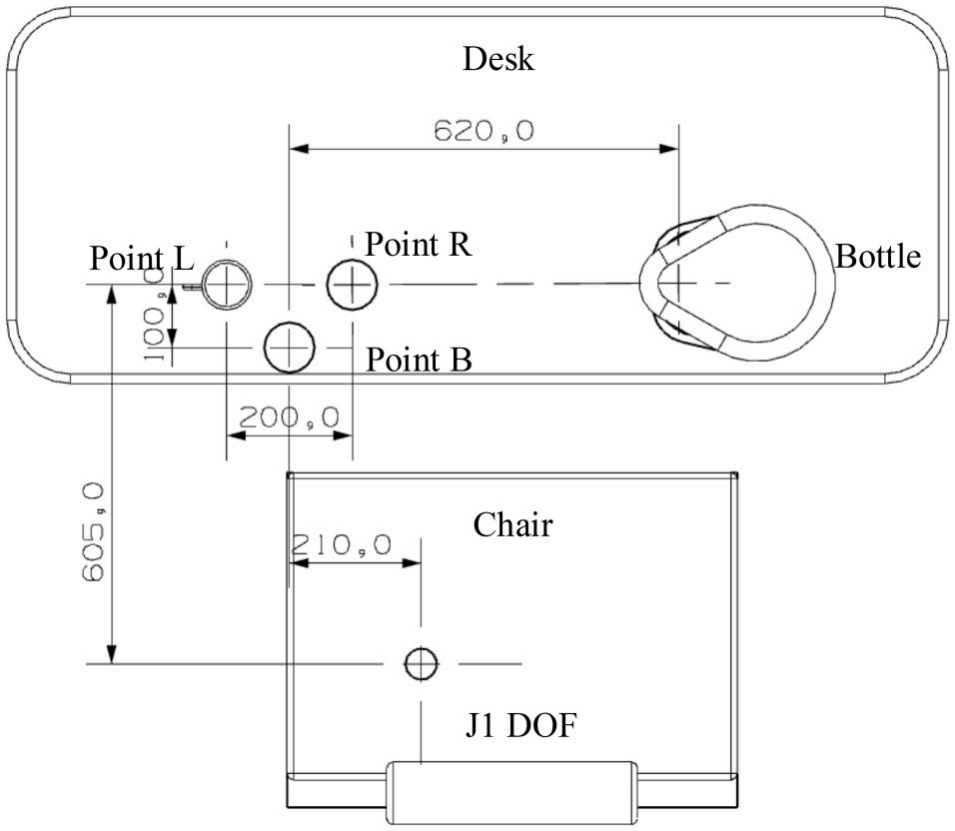
**Positions of three cup points are designed as a triangle on a plane**.

According to the first principle, three cup points are set up for P2, which leads to different movement trajectories during A2 and A3 actions. Three cup points were set up, as shown in Figure 4, including the left point (Point L), right point (Point R), and bottom point (Point B). The locations of the chair, the desk, the bottle, and J1 DOF are also fixed.

In the experiment, movement parameters for different cup points were recorded. At first, subjects were instructed to perform the task for one cup point without any practice. After practicing, they were instructed to perform it at the same point again. This point was chosen as the left point. Then, subjects proceeded to complete the task with random cup points assigned by the researcher.

### Task description

**Figure 5:**
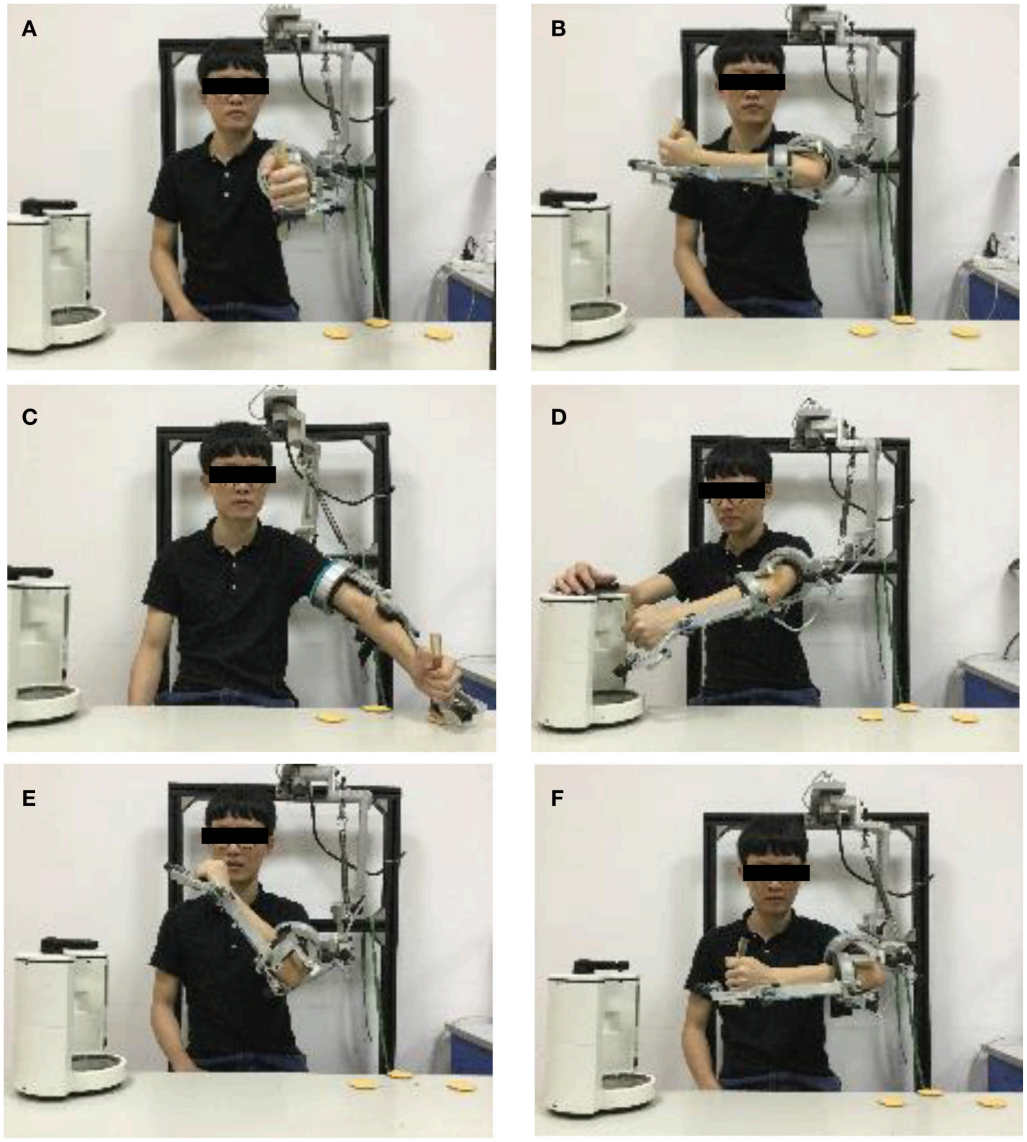
**Movement design**. **(A)** P0 posture. **(B)** P1 posture. **(C)** P2 posture. **(D)** P3 posture. **(E)** P4 posture. **(F)** P5 posture. A1 action is the motion from P0 to P1; A2 action is the motion from P1 to P2; A3 action is the motion from P2 to P3; A4 action is the motion from P4; A5 action is the motion from P4 to P5.

According to the second and the third principles, a series of movements for drinking was proposed in the experiment. The subject had to use two hands coordinating with each other. Neo-Arm I was worn on the left upper limb to perform a series of movements, including five actions and five hovering postures, as shown in Figure 5: zero position posture (P0), ready action (A1), ready posture (P1), fetching cup action (A2), holding cup posture (P2), getting water action (A3), getting water posture (P3), drinking action (A4), drinking posture (P4), homing action (A5), and homing posture (P5).

The first posture (P0) is zero position posture, as shown in Figure 5A, which is a fixed initial posture when the subjects put on the device in the task. P1 is the position when subjects get ready to fetch the cup, which is shown in Figure 5B. A1 is the action in which the upper limb is moved from P0 to P1. A2 is the action in which subjects move the upper limb to fetch the cup. Although, it is essential that the subject completes the action of picking up the cup in real-world situations, hand function training is not the focus of this paper. Therefore, the catching action is simplified as moving the handle of the device and reaching the cup point. The posture where the upper limb hovers at the cup point is P2, as shown in Figure 5C. Then, the subject should move the left upper limb from the cup point to the bottle through A3 action. At P3, although the right upper limb is not tested in this research, the subject has to use his/her right hand to press the button to coordinate with the motion of the left upper limb, shown as in Figure 5D. After that, the subject should move the handle from the bottle to the side of his/her mouth and perform the drinking movement through the A4 action. Meanwhile, the right side of the upper limb must help to hold the cup. Finally, the upper limb should be moved to the ready posture.

## Results

### ROM

**Figure 6:**
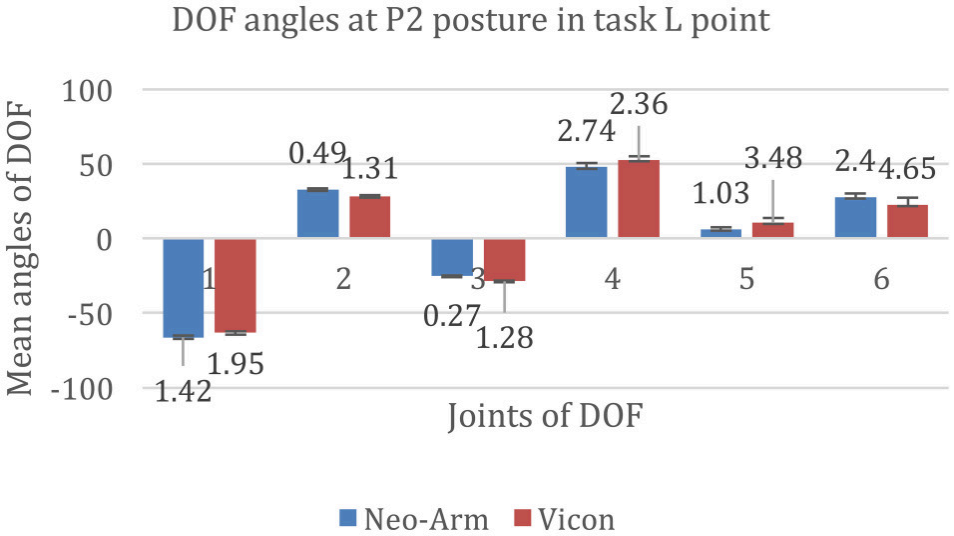
**DOF angles at P2 at the L point in the Point Left task**. 1, 2, 3, 4, 5, and 6 represent the angles of J1, J2, J3, J4, J5, and J6 in the Point Left task.

**Figure 7:**
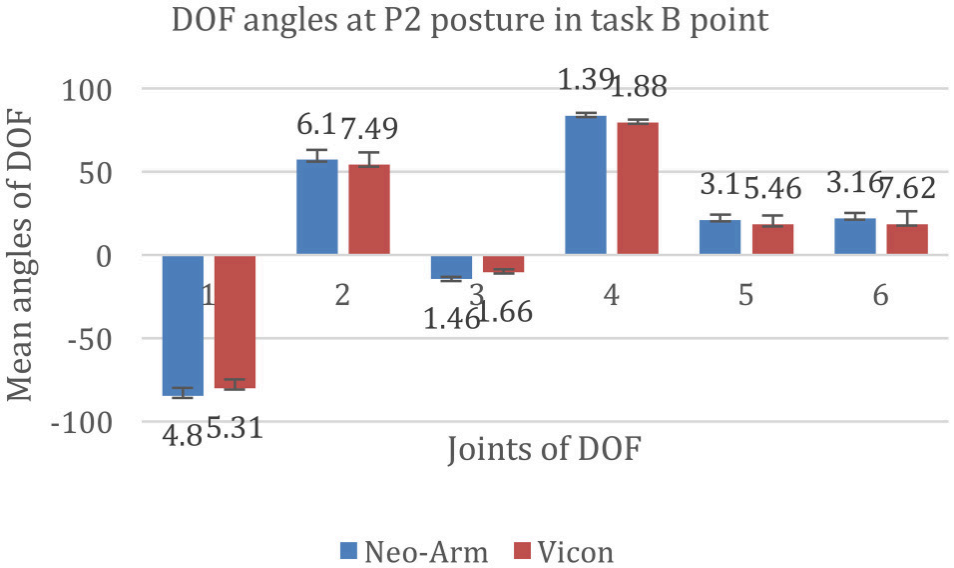
**DOF angles at P2 at the B point in the Point Left task**. 1, 2, 3, 4, 5, and 6 represent the angles of J1, J2, J3, J4, J5, and J6 in the Point Bottom task.

**Figure 8:**
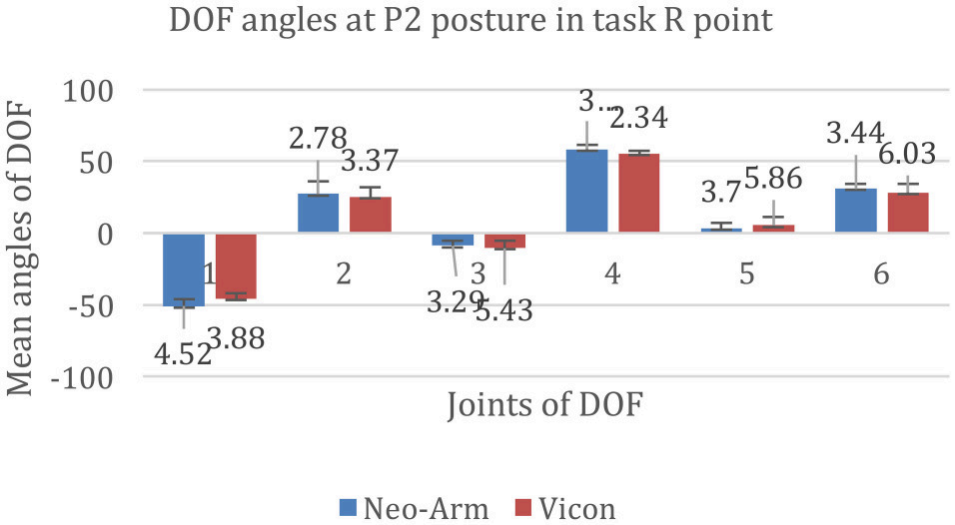
**DOF angles at P2 for the R point in the Point Right task**. 1, 2, 3, 4, 5, and 6 represent the angles of J1, J2, J3, J4, J5, and J6 in the Point Bottom task.

**Figure 9:**
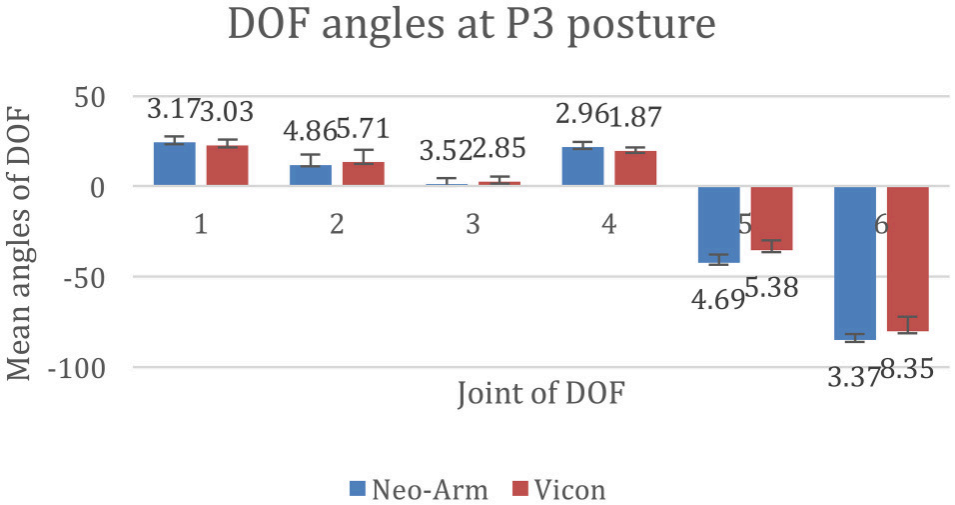
**DOF angles at P3**. 1, 2, 3, 4, 5, and 6 represent the angles of J1, J2, J3, J4, J5, and J6.

**Figure 10:**
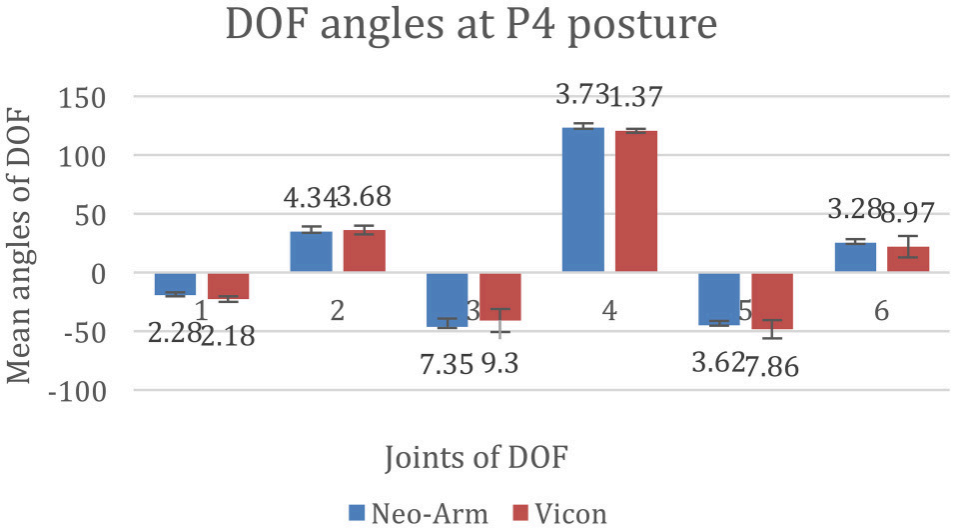
**DOF angles at P4**. 1, 2, 3, 4, 5, and 6 represent the angles of J1, J2, J3, J4, J5, and J6.

According to the different periods of the whole movement, the movement in the task was divided into five actions and six hovering postures. Since the reaching cup motion, the getting water motion, and the drinking motion play important roles in the task, the angle of each DOF from all subjects at the P2, P3, P4, and P5 postures are listed in Figures 6–**10**.

The data for P2 at the L point are shown in Figure 6. The mean angles of J1 from Neo-arm and Vicon are −66.28 ± 1.42 and −63.26 ± 1.95 mm (*p* = 0.038); the mean angles of J2 are 32.96 ± 0.49, 28.50 ± 1.31 mm(*p* = 0.046); the mean angles of J3 are −25.14 ± 0.27, −28.48 ± 1.28 mm(*p* = 0.031); the mean angles of J4 are 47.84 ± 2.74, 52.65 ± 2.36 mm(*p* = 0.042); the mean angles of J5 are 6.21 ± 1.03, 10.56 ± 3.48 mm(*p* = 0.11); the mean angles of J6 are 27.78 ± 2.4, 22.65 ± 4.65 mm(*p* = 0.12).

The data for P2 at B point are shown in Figure 7. The mean angles of J1 from Neo-arm and Vicon are −84.86 ± 4.8, −80.26 ± 5.31 mm (*p* = 0.062), the mean angles of J2 are 57.16 ± 6.1, 54.19 ± 7.49 mm (*p* = 0.053), the mean angles of J3 are −14.55 ± 1.46, −12.33 ± 1.66 mm (*p* = 0.037), the mean angles of J4 are 83.89 ± 1.39, 79.67 ± 1.88 mm (*p* = 0.046), the mean angles of J5 are 21.06 ± 3.1, 18.35 ± 5.46 mm (*p* = 0.032), the mean angles of J6 are 22.04 ± 3.16, 18.61 ± 7.62 mm (*p* = 0.067).

The data for P2 at the R point are shown in Figure 8. The mean angles of J1 from Neo-arm and Vicon are −50.86 ± 4.52, −45.64 ± 3.88 mm (*p* = 0.055), the mean angles of J2 are 27.26 ± 2.78, 25.37 ± 3.37 mm (*p* = 0.036), the mean angles of J3 are −8.86 ± 3.29, −10.50 ± 5.43 mm (*p* = 0.044), the mean angles of J4 are 58.30 ± 3.11, 55.16 ± 2.34 mm (*p* = 0.036), the mean angles of J5 are 3.37 ± 3.70, 5.35 ± 5.86 mm (*p* = 0.067), the mean angles of J6 are 28.16 ± 6.03, 31.00 ± 3.44 mm (*p* = 0.072).

The data for P3 posture are shown in Figure 9. The mean angles of J1 from Neo-arm and Vicon are 24.36 ± 3.17, 22.67 ± 3.03 mm (*p* = 0.028), the mean angles of J2 are 11.91 ± 4.86, 13.38 ± 6.71 mm (*p* = 0.043), the mean angles of J3 are 1.07 ± 3.52, 2.55 ± 2. 88 mm (*p* = 0.035), the mean angles of J4 are 21.63 ± 2.96, 19.57 ± 1.97 mm (*p* = 0.038), the mean angles of J5 are −42.28 ± 4.69, −35.38 ± 5.68 mm (*p* = 0.24), the mean angles of J6 are −85.20 ± 3.37, −80.36 ± 8.35 mm (*p* = 0.14).

The data for P3 are shown in Figure 10. The mean angles of J1 from Neo-arm and Vicon are −19.20 ± 2.28, −22.13 ± 2.18 mm (*p* = 0.041), the mean angles of J2 are 34.84 ± 4.34, −22.13 ± 2.18 mm (*p* = 0.032), the mean angles of J3 are −46.32 ± 7.35, −40.35 ± 9.3 mm (*p* = 0.058), the mean angles of J4 are 123.28 ± 3.73, 120.89 ± 1.37 mm (*p* = 0.044), the mean angles of J5 are −44.39 ± 3.62, −48.32 ± 7.86 mm (*p* = 0.13), the mean angles of J6 are 25.56 ± 3.28, 22.13 ± 8.97 mm (*p* = 0.17).

Considering the differences among subjects, the average and the median angles of each DOF are listed in Table 6.

**Table 6:** **Median and average angles of each DOF in three points of the task**.

		**J1**	**J2**	**J3**	**J4**	**J5**	**J6**
P2	Median (L)	−66.75	32.34	−25.04	44.97	4.3	28.9
	Avg (L)	−66.57	32.11	−25.42	45.26	4.49	28.87
	Median (R)	−50.02	28.61	−6.82	59.7	1.71	28.37
	Avg (R)	−50.15	28.84	−6.69	60.08	2.12	28.62
	Median (B)	−86.63	56.55	−15.32	83.84	20.24	20.92
	Avg (B)	−85.75	55.86	−15.24	83.62	20.12	21.13
P3	Median	23.82	11.74	−0.02	22.12	−42.16	−85.38
	Avg	24.22	11.85	0.15	21.7	−42.2	−85.12
P4	Median	−19	35.06	−46.26	123.36	−44.02	25.71
	Avg	−19.2	34.84	−46.32	123.28	−44.39	25.56

### Velocity

In the experiment, during each action, the average angular velocities are listed in Table 7. During the A2 and A3 periods, since different cup points lead to different actions, they are listed with v_L, v_R, and v_B, which represent the velocity of different actions for A2 and A3.

**Table 7:** **Velocity of each joint during each motion**.

		**J1**	**J2**	**J3**	**J4**	**J5**	**J6**
A1		0.00	0.00	0.00	89.53	−6.68	78.47
A2	v_L	−69.73	25.73	−22.02	70.99	10.15	37.27
	v_R	−119.15	53.83	−9.64	54.44	28.39	63.13
	v_B	−157.28	113.35	−27.40	38.92	27.15	75.19
A3	v_L	−90.79	20.26	−25.57	23.57	46.69	113.99
	v_R	−74.37	16.99	−6.84	38.39	44.32	113.74
	v_B	−109.98	44.01	−15.40	61.92	62.32	106.25
A4		−43.43	22.99	−46.47	101.59	−2.18	110.68
A5		26.10	−45.65	67.71	−31.21	67.51	99.78

### Measured ROM

**Figure 11:**
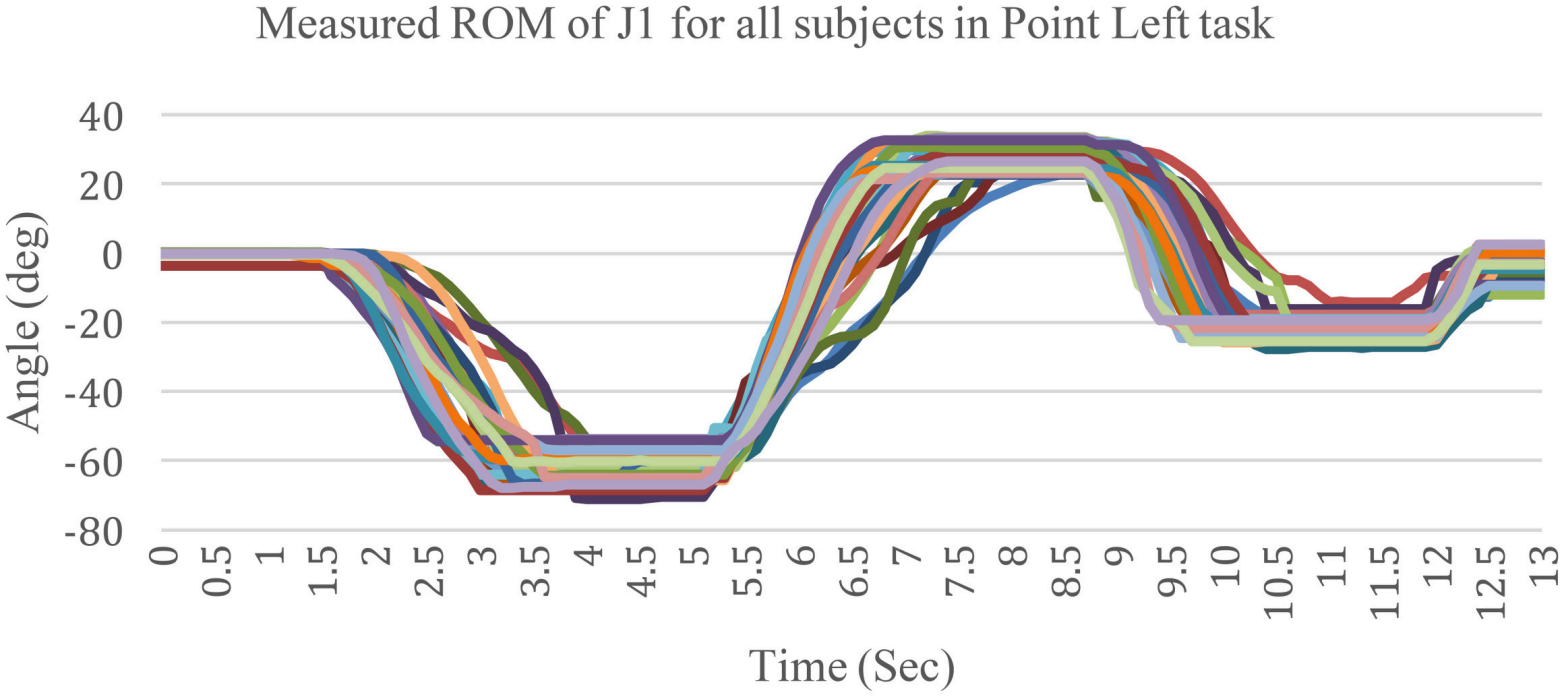
**Measured ROM of J1 for all subjects in the Point Left task**.

Figure 11 shows the motion of J1 from eight subjects in the Point Left task. J1 represents the shoulder abduction/adduction. To compare all curves, the suspending period has been adjusted within 2 s. Besides, the other five DOFs were solved with the same manner as the J1 DOF.

### Movement scheme

**Figure 12:**
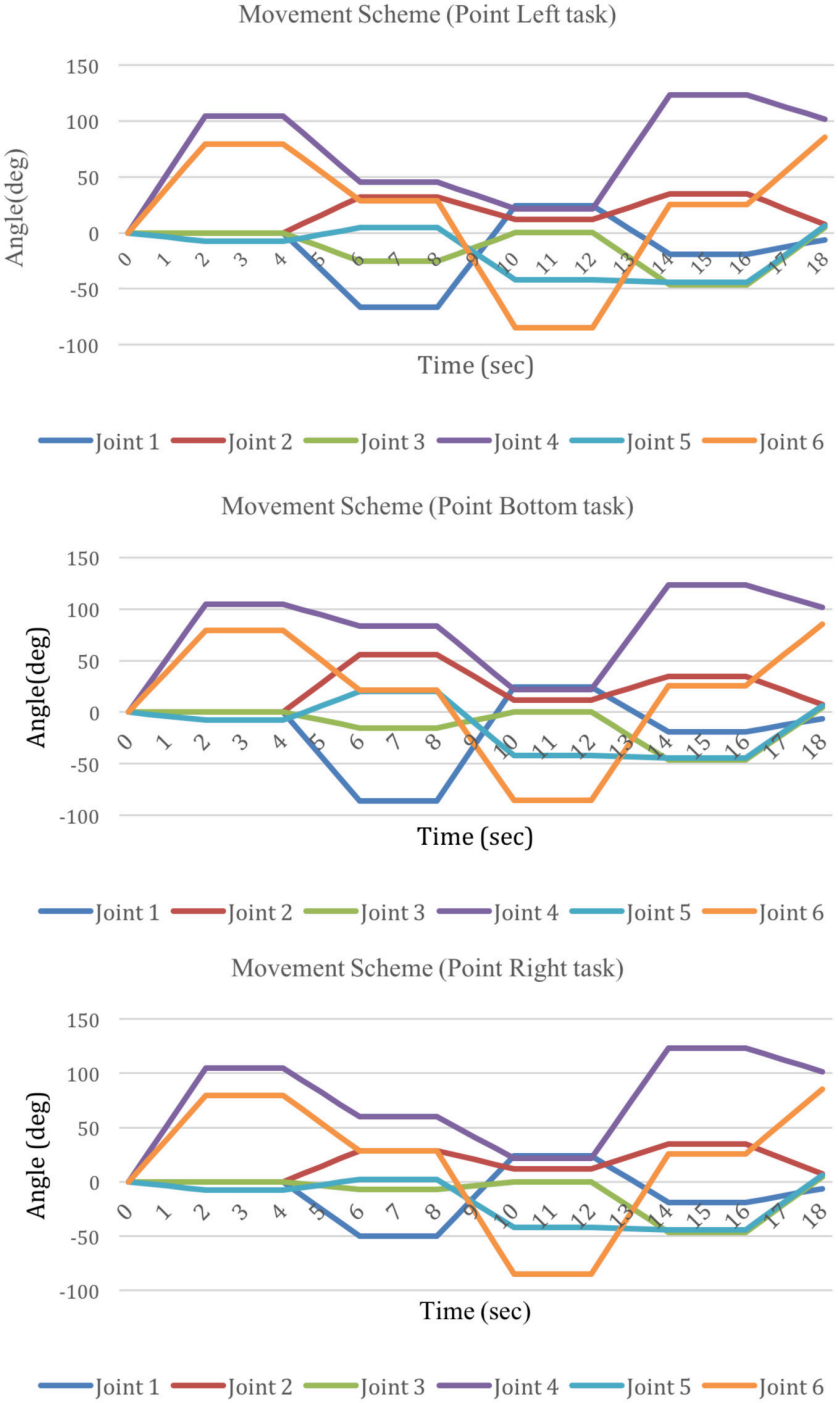
**Movement scheme for the Point Left, Point Bottom, and Point Right tasks**.

Since the average angular and the average velocity have been obtained, the movement scheme is shown in Figure 12. It is shown that the angle of each joint, which is displayed as a distinctive color, varies with time. Meanwhile, since the velocity of the endpoint cannot reflect the whole movement comprehensively, the velocity scheme is proposed, as represented by the gradient of each line.

### 3D trajectory

**Figure 13:**
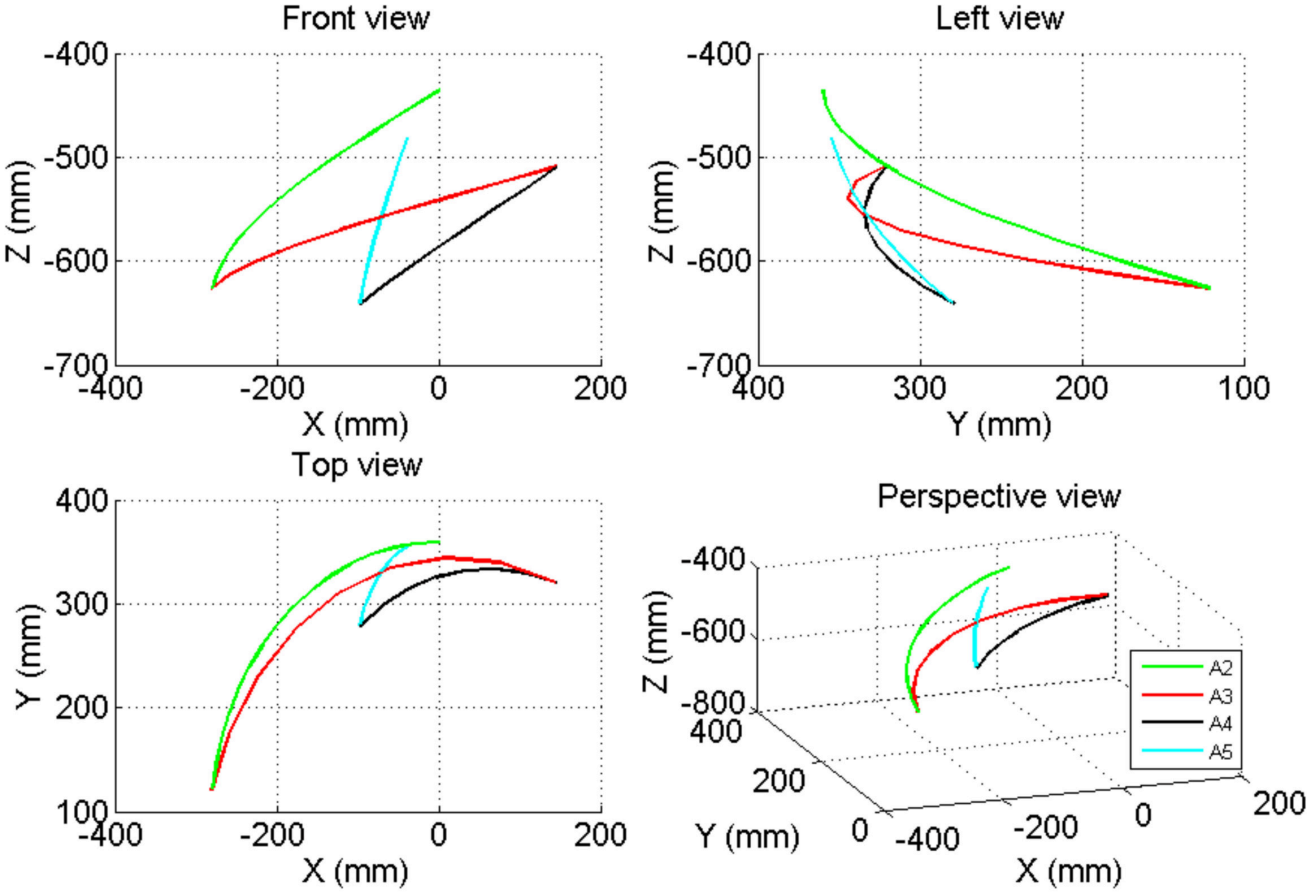
**The 3-D trajectory of the movement of the elbow joint in the Point Left task**.

**Figure 14:**
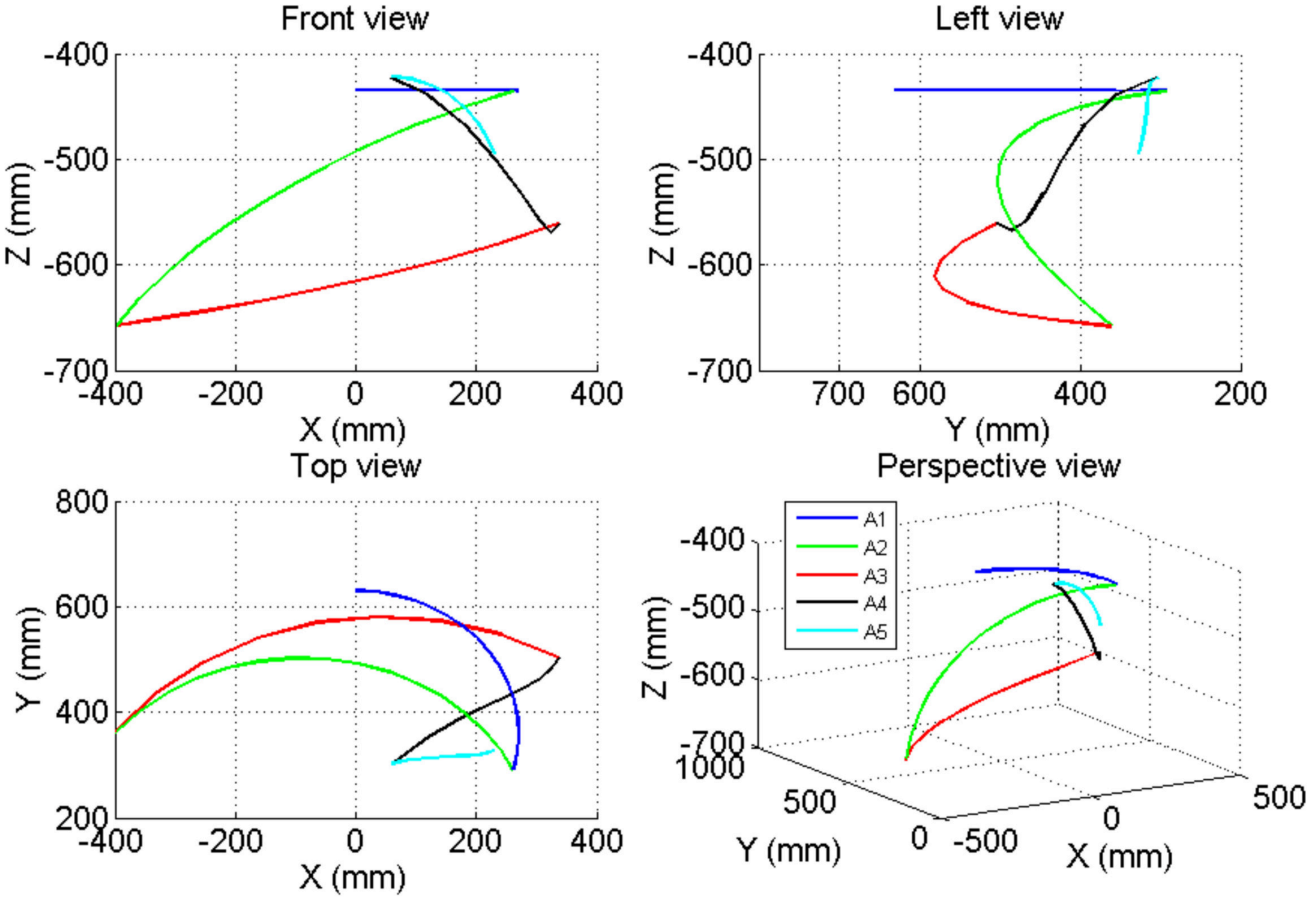
**The 3-D trajectory of the movement of the wrist joint in the Point Left task**.

According to the movement scheme, the 3D trajectory is shown in Figures 13, 14. Considering that the movement of the wrist would reflect the whole movement trajectory with interference, the intersection of the forearm pronation/supination axis and wrist flexion/extension axis was chosen as the endpoint. The y-z plane represents the horizontal plane. The zero point represents the ending point of the upper limb when it is at the zero posture position, the cup point represents the position where the cup is to be placed, the bottle point represents the position where the water bottle is to be placed. The segment of line 1 represents the movement of reaching for the cup, the segment of line 2 represent the movement of moving the cup to the water bottle, and the segment of line 3 represents the movement of drinking.

## Discussion

### Comparison between Neo-Arm and vicon

**Figure 15:**
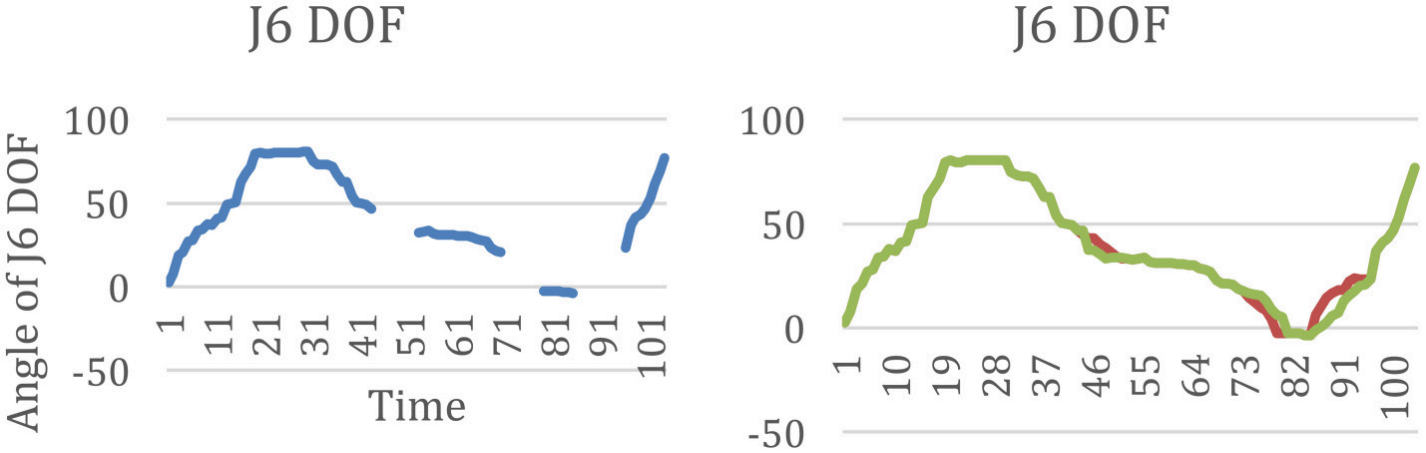
**(A)** The lost points of J6 DOF in the movement before simulation from Vicon, **(B)** the two results of the simulation motion data of J6 from Vicon.

When the hand reached the P2 posture for the cup and P3 for the bottle, the marks on the wrist and the hand were blocked by the cup and the bottle. In this situation, the marks in the Vicon software are lost in these zones, see Figure 15A. To generate a continuous trajectory, the Vicon polygon software simulated the motions of the lost mark based on the trajectory captured previously and afterward. Meanwhile, when one mark is blocked, the Vicon polygon system can still calculate the results from the motion data of adjacent marks. For example, in the two simulated trajectories, J6 DOF varied in two ways, see Figure 15B. According to the simulation, both ways are reasonable. However, during this process, the simulated values of the angles varied for different trajectories, which resulted in a larger standard deviation and *p* > 0.05 for three DOF of the wrist in the Vicon system than in Neo-Arm.

### Motion coordination and ROM for the point left task

According to Figure 12, there are similar patterns in three points tasks. Especially in the shoulder joints and the elbow, four DOFs change with time in a similar pattern.

During the A2 period, in the first half, J1 rotated from the zero position posture to the left side, with a maximum of −66.57° on average, while J3 started to increase from 0° at a low velocity. In the second half of A2, the angle displacement of J3 increased at a high velocity, while J4 rotated from the maximum to the minimum. The changes in this period mean that when subjects moved their left arm to reach the cup, which was placed on the left side, they were inclined to perform shoulder abduction first, accompanied by slight shoulder external rotation synchronously. After that, subjects tried to press their elbow by the way of shoulder extension, while the elbow extended at a maximum angle to open their arm. However, the motion of J5 exhibited two patterns. Subjects tended to pronate the forearm to 4.3° on average. By this way, the wrist is adjusted to perform radial deviation, so the subjects could move J5 in the tangential direction of the space trajectory.

During A3, the angular variation of J2 and J3 led the arm move in advance. The two joints started to rotated back to ~0°. During the second half of this process, J1 started to increase to a maximum angle of 23.82° on average, while J4 rotated to 22.12° on average. J5 moved to an angle of −42.16 on average. To perform this action, subjects first lifted their elbow using shoulder flexion and internal rotation. Before they finished the lifting, the shoulder began to adduct, accompanied by elbow flexion. All these variations stopped simultaneously when the hand reached the water bottle position. The forearm and the wrist remained still in this process.

During A4, J1 decreased at a high velocity while J4 increased at a high velocity. In this process, J3, J5, and J6 increased slightly. In this process, the elbow flexed while the shoulder abducted. By this movement, subjects moved the hand to the mouth position. At the same time, the forearm pronated to maintain a drinking gesture.

### Comparison between two patterns of variations of velocity

**Figure 16:**
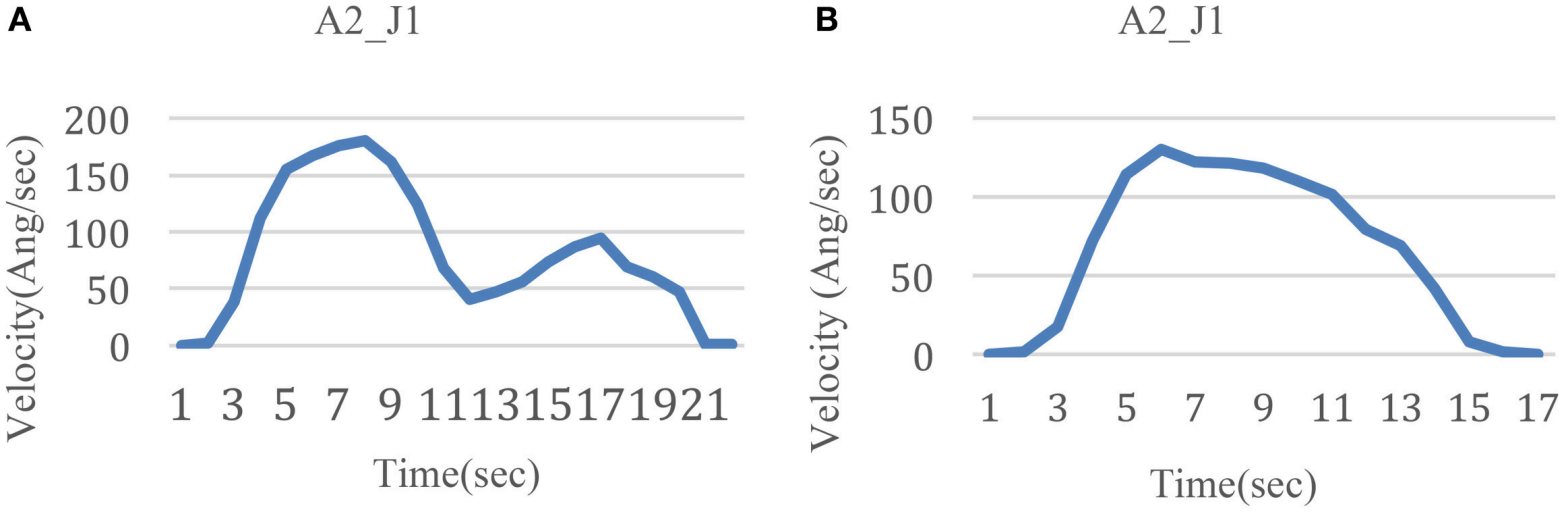
**Two modes of velocity variation**. **(A)** M-curve for J1 during A2 **(B)** N-curve for J1 during A2.

During A2 and A3, two major modes of angular velocity curves were presented for J1, referred to as the M-curve and N-curve, as shown in Figure 16. The major difference between these two types of curves is that there are two wave crests in the M-curve, between which there is a wave trough. Meanwhile there is only one wave crest in the N-curve.

In the experiment, this difference involves three kinds of conditions. The first is whether the subjects have practiced with the device and the task. When subjects performed the fixed cup point task without any practice, the angular velocity varied in the M-curve mode. Meanwhile, the angular velocity varied in the N-curve mode after subjects experienced the practice. The reason for this phenomenon is that the practice made subjects more skillful in the task. The more skilled the subjects became, the more smoothly they moved.

Second, even after practice, when the testing mode was changed from a fixed cup point to random cup points, the angular velocity curve appeared to be in the M-curve mode. This is because compared with the fixed cup point task, mechanical repetition was avoided. And the more familiar they became with the task, the less their conscious thought process was involved. However, when performing the random cup point task, subjects had to focus on the task goal and made corresponding movement adjustments. As such, the random cup point task motivated their conscious thought process more than the fixed cup point.

Third, this difference occurred obviously in J1 and J4 during A2 and A3. This is because horizontal movements played a main role in the two actions. The range of abduction and adduction of J1 for the shoulder was large. Other movements of joints were also accompanied during the same period, which lead to a reduction in the movement smoothness.

### Comparison between the three points

The functional movement in daily life is not limited in fixed posture and motion. In order to provide a task with different range of motion (ROM), we proposed three cup points in this research. Through the three points, the subject need to move their upper limb in different ROM. At each point, the posture of the upper limb is distinctive. In the task, according to the different points, it is shown that the angle of each DOF is distinctive at each posture, see Figures 6–10.

Meanwhile, when the task is set with one cup point, the subject can be familiar with the task soon, and form a mechanical movement, without the active consciousness. But in the task of random points mode, the velocity of DOF varied in a M-curve type, see Figure 16. It is considered that this velocity variation mode is caused by the active consciousness from the subject. So, the three points task is one solution for the upper limb active training. In this case, the three points movement scheme should be obtained from the research, see Figure 12.

### Movement scheme

For Point Left task, the time length of the period of hovering is set at two seconds. During this time interval, all the joints should be kept still. This period can be prolonged as required. But it is still necessary that each interval last for at least 2 s. This time period is set for the subject to adjust his/her movement. Since A1 is for reparation and A5 is for backing, which includes simple rotation for DOF, it is detailed discussed on A2, A3, and A4 as below.

In the scheme, A2 lasts for 2 s. The objective is to open the upper limb to reach a cup placed on the left side. This action is designed in two parts. For 4–6 s, the shoulder extends slightly at a low velocity. The shoulder abduction is accomplished within the first 1 s, and the forearm pronation is accomplished within the first 0.5 s. This forearm pronation is needed to adjust the wrist at an angle by which the fist can move along the tangential direction of the space trajectory. In this way, the forearm can move more easily and fluently. The elbow merely extends slightly, while the forearm follows the shoulder extension. For 6–8 s, the shoulder abduction stops at the maximum angle. The elbow is pressed down as a result of shoulder external rotation and extension, which is accompanied by wrist radial deviation. At the same time, the elbow extends with vast angular displacement at a high velocity. It is obvious that during the A2 period, shoulder external rotation and elbow extension occur near the end of shoulder abduction.

A3 lasts for 2 s. First, for 8–10 s, the elbow moves up with the assistance of the gravity compensation mechanism by means of shoulder flexion and internal rotation. Meanwhile, the elbow has slight flexion. Then, for 9–10 s, the shoulder accomplishes the adduction swiftly while the elbow finishes the remaining part of the flexion. A4 lasts for 12–14 s. During this period, the shoulder abducts greatly and the elbow flexes to move the hand to the mouth position to maintain a drinking gesture. The forearm pronates at an angle to perform the gesture of maintaining the cup.

This movement scheme can be stored as a training task on a computer and applied for upper limb rehabilitation training with exoskeleton robots.

## Conclusion

In the research, subjects were instructed to move the left upper limb from the center at the front of the chest to the left side, and then from the left side to the right side, and then back to the body. During these movements, subjects were inclined to adjust the upper arm to lead the distal joints when the task was to move the upper limb from a proximal to a distal location. Meanwhile, subjects were inclined to adjust the distal joints when the task was to move the upper limb from a distal to a proximal location.

Motion data were obtained using two systems, Neo-Arm and Vicon. The motion of each DOF of the upper arm was directly captured by Neo-Arm, while with Vicon, it was calculated by the trajectories of reflective marks. The results indicate that the Neo-Arm can achieve the suitable precision and be fit for other kinds of upper limb motion tasks. The motion data of three points in task are captured and stored as a scheme in the task library on a computer.

## Author contributions

LL: main author who have did this research and wrote the manuscript. LX: the doctoral supervisor of the LL and YS, who proposed the research and direct us in the experiment. YS: assist in the experiment and part of work in data processing. BA: proposed the opinions from the medicine, and proposed the control experiment.

### Conflict of interest statement

The authors declare that the research was conducted in the absence of any commercial or financial relationships that could be construed as a potential conflict of interest.
